# Supplementary figures and images for: Age and micronutrient effects on the microbiome in a mouse model of zinc depletion and supplementation
Source: PLoS One. 2022 Dec 19;17(12):e0275352. doi: 10.1371/journal.pone.0275352 (PMC9762596; doi:10.1371/journal.pone.0275352)

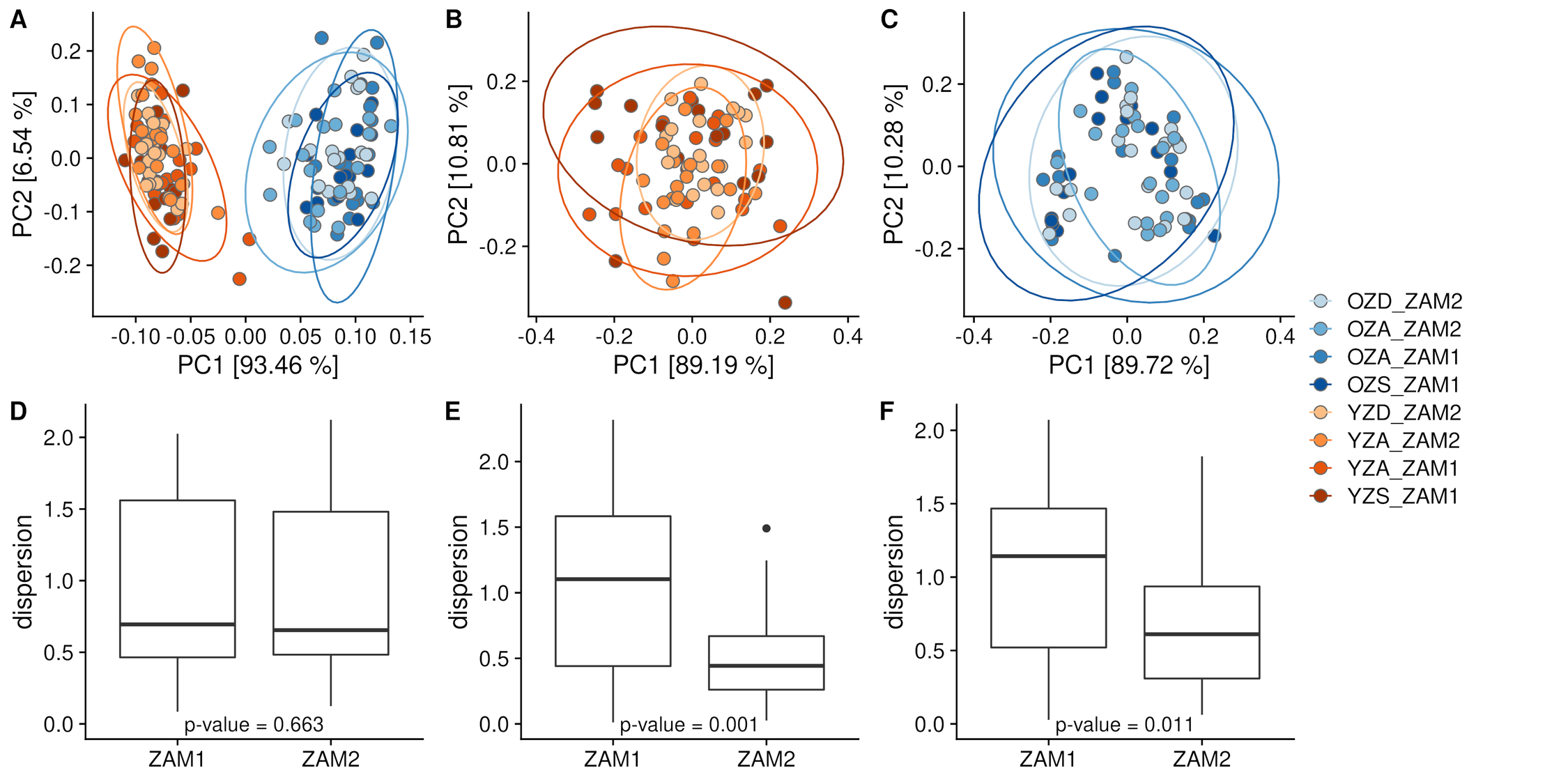

Supplement: S1 Fig — Robust principal component analysis results are shown for combined ages, young mice and old mice, respectively (A-C). The intra-study dispersions are shown using boxplots (D-F) for combined ages, young mice, and old mice, respectively, with the first, median, and third quartiles shown by the hinges, and the whiskers extend to no more than 1.5 x inter-quartile range, with outliers plotted individually. (TIF) [file pone.0275352.s002.tif]
